# Impact of Cataract Surgery on Higher Order Aberrations and Comparative Analysis using Scheimpflug imaging and ray-tracing technology

**DOI:** 10.22336/rjo.2025.11

**Published:** 2025

**Authors:** Deepti Saxena, Vikas Kanaujia, Ankita Ranjan, Vaibhav Jain, Ankita Aishwarya, Ahmad Husain, Rachna Agarwal

**Affiliations:** Department of Ophthalmology, Sanjay Gandhi PostGraduate Institute of Medical Sciences, Lucknow, Uttar Pradesh, India

**Keywords:** cataract, higher-order aberrations (HOAs), Scheimpflug imaging, ray-tracing technology, HOA = higher-order aberrations, RMS = root mean square, LOA = Lower-Order aberration, IOL = Intraocular lens, BCVA = best-corrected visual acuity

## Abstract

**Objective:**

This study aims to evaluate higher-order aberrations (HOAs) pre- and post-cataract surgery using advanced technologies to achieve an acceptable best-corrected visual outcome in favor of patients.

**Methods:**

This prospective, observational study involves 200 adult patients (aged 40 years or older) undergoing cataract surgery. All these patients have been evaluated before and after cataract surgery.

The study involved comprehensive measurements of higher-order aberrations (HOAs) using both Scheimpflug imaging (Sirius system) and ray-tracing technology (iTrace). These measurements were taken preoperatively and at specific postoperative time points (7 days, 1 month, and 2 months). Various parameters were analyzed, including the root mean square (RMS) of total HOAs and specific corneal HOAs. Patients were followed up for 2 months postoperatively.

**Results:**

We found no significant differences between the S and I methods at any time point for lower-order aberration (LOA). The S method generally measured higher LOA values than the I method. Significant differences were observed between the S and I methods at all time points for HOA. The S method consistently measured higher HOA values. The S method showed small but significant changes over time, while the I method showed no significant changes.

**Discussion:**

This study provides comprehensive insights into the changes in corneal curvature and higher-order aberrations following modern cataract surgery. The findings suggested that while small changes occur in corneal properties, these have minimal impact on visual outcomes. The significant improvement in vision appears to be primarily driven by replacing the cataractous lens with an IOL, particularly in reducing total ocular aberrations.

**Conclusion:**

Our study highlighted a significant reduction in total ocular higher-order aberrations (HOAs), particularly spherical aberrations. This reduction is a key factor in improving visual quality after cataract surgery. This reduction is primarily attributed to replacing the cataractous lens with an IOL rather than to changes in corneal optics.

## Introduction

Cataract surgery has undergone significant evolution over the past few decades, transforming from a sight-restoring procedure to a refractive surgery aimed at optimizing visual outcomes [1]. As the leading cause of reversible blindness worldwide, cataracts affect millions of people, particularly in the aging population [1]. With increasing life expectancy and the rising importance of maintaining high-quality vision for work and daily activities in the elderly, the demand for excellent visual outcomes following cataract surgery has never been higher.

The cornea, responsible for approximately two-thirds of the eye’s total refractive power, is crucial in determining visual quality after cataract surgery. Traditionally, cataract surgery focused primarily on removing the cloudy lens and implanting an intraocular lens (IOL) to restore basic visual function [2,3]. However, modern cataract surgery has shifted toward a more comprehensive approach, considering various factors that influence postoperative visual outcomes, including corneal curvature and higher-order aberrations (HOAs) [1,2].

Higher-order aberrations, which traditional spherocylindrical lenses cannot correct, have gained increasing attention in recent years [2]. These subtle optical imperfections can significantly impact visual quality, affecting contrast sensitivity, glare, and overall visual satisfaction. Cataract surgery can alter corneal HOAs through direct effects on corneal structure or indirectly through changes in the eye’s optical system following IOL implantation. HOAs are typically described using Zernike polynomials, with the most clinically relevant being coma, trefoil, and spherical aberration [1]. These aberrations can cause visual symptoms such as glare, halos, and reduced contrast sensitivity, even in eyes with excellent visual acuity as measured by standard charts.

In the context of cataract surgery, HOAs are significant for several reasons. The cornea is a primary source of ocular HOAs. Preexisting corneal HOAs can significantly influence postoperative visual quality and must be considered in surgical planning [4]. The cataract surgical procedure itself can induce changes in corneal HOAs. Factors such as incision location, size, and construction technique can all influence postoperative aberrations [5]. The implanted intraocular lens can introduce its aberrations or modify existing ones. The design and positioning of the IOL are crucial factors in managing postoperative HOAs [6]. As the eye ages, there are natural changes in HOAs, particularly an increase in spherical aberration. Cataract surgery provides an opportunity to address these age-related changes [7].

Numerous studies have examined the impact of HOAs on visual quality after cataract surgery. Research has shown that even small amounts of HOAs can significantly affect visual performance, particularly in low-light conditions or when performing visually demanding tasks [8]. The management of HOAs in cataract surgery extends beyond just spherical aberration. Coma and trefoil are also essential considerations induced by decentered or tilted IOLs. Precise IOL positioning and careful surgical technique are crucial for minimizing these aberrations [9].

The measurement and analysis of HOAs have also advanced significantly. Based on Hartmann-Shack technology, wavefront aberrometry allows for the detailed quantification of ocular aberrations [10]. More recently, ray-tracing aberrometry has emerged as a promising technique, offering potential advantages in accuracy and the ability to measure highly aberrated eyes [11].

Despite these advancements, managing HOAs in cataract surgery still faces several challenges, including variability in pupil size, neural adaptation, individual variations, predicting postoperative aberrations, adaptive optics, multifocal IOLs, and HOAs [12].

In conclusion, managing higher-order aberrations has become an integral part of modern cataract surgery. As our understanding of HOAs and their impact on visual quality continues to grow, so does our ability to address them effectively [12]. Future advancements in this field will likely refine our approach to HOAs, potentially leading to even better visual outcomes for cataract surgery patients.

This study examines changes in and higher-order aberrations before and after cataract surgery, utilizing Scheimpflug and ray-tracing principles. By comparing preoperative and postoperative measurements, we sought to gain a deeper understanding of how cataract surgery impacts these key components of visual quality. This knowledge will contribute to the evolution of cataract surgery toward a more precise and personalized approach, ultimately leading to improved visual outcomes and patient satisfaction.

## Material and methods

This study employed a prospective, observational design to evaluate changes in higher-order aberrations (HOAs) before and after cataract surgery using Scheimpflug imaging and ray-tracing technologies. The study was conducted over 18 months (January 2023 to June 2024), incorporating multiple assessment time points to capture both short-term and medium-term changes in corneal properties and optical quality. Measurements were taken preoperatively and then postoperatively at 7 days, 1 month, and 2 months postoperatively.

The study population consisted of adult patients diagnosed with cataracts and scheduled for elective cataract surgery at the institution. Patients aged 40 years or older with cataracts up to grade 3 as per the Lens Opacities Classification System III (LOCS III) and those scheduled for elective cataract surgery were included in this study. History of any ocular disease (other than cataract), previous history of any eye surgery, pre-op corneal astigmatism more than 0.75 diopter, inability to dilate pupil ≥ 6 mm, risk of posterior capsule rupture as in posterior polar cataract, lens dislocation, high myopia > -9D, ocular conditions that could confound corneal HOAs (e.g., amblyopia, ocular surface inflammation, dry eye disease, keratoconus, corneal dystrophies, intraocular inflammation) and patients who refused to participate in the study were excluded from this study.

The sample size was calculated based on a previous study by Guirao A et al. (2004), which reported changes in root mean square (RMS) values between preoperative and postoperative measurements. Assuming an effect size of 0.25, a two-sided 95% confidence interval, and 90% power, the estimated sample size for paired group analysis was 171. To account for potential dropouts and ensure adequate statistical power, the target sample size was set at 200 patients.

The study involved comprehensive measurements of HOAs using both Scheimpflug imaging (Sirius system) and ray-tracing technology (iTrace). These measurements were taken preoperatively and at specific postoperative time points (7 days, 1 month, and 2 months).

All patients underwent a comprehensive preoperative evaluation, including:

1. Medical history and systemic workup (vitals, complete blood count, fasting blood sugar, HbA1c, viral markers);
2. Visual acuity assessment (uncorrected using logMAR values and best-corrected);
3. Intraocular pressure measurement (Goldmann Applanation Tonometry);
4. Slit-lamp biomicroscopy;
5. Fundoscopy;
6. IOL power calculation using IOL master 700;
7. Corneal topography and aberrometry using Scheimpflug imaging (Sirius system);
8. Total ocular aberrometry using ray-tracing technology (iTrace).

Scheimpflug imaging was performed first to eliminate preference bias, followed by ray-tracing aberrometry for all patients.

All patients underwent standard phacoemulsification cataract surgery on the Centurion Phaco machine, manufactured by Alcon. This highly advanced device features a 2.2 mm precise corneal incision and implants a foldable, hydrophobic, aspheric or spheric intraocular lens (IOL). The choice between aspheric and spheric IOLs was based on the preoperative Q value and spherical aberration of the cornea to minimize bias in postoperative aberration measurements. All surgeries were performed by experienced surgeons following a standardized technique.

Postoperative evaluations were conducted 7 days, 1 month, and 2 months after surgery. Each follow-up visit included:

1. Visual acuity assessment;
2. Slit-lamp examination;
3. Intraocular pressure measurement;
4. Corneal topography and aberrometry using Scheimpflug imaging;
5. Total ocular aberrometry using ray-tracing technology.

The study analyzed various parameters, including corneal curvatures (K1, K2, Km), astigmatism, the root mean square (RMS) of total higher-order aberrations (HOAs), and specific corneal HOAs. It assessed the best-corrected visual acuity (BCVA) to measure functional visual outcome.

The study was conducted in accordance with the Declaration of Helsinki and was approved by the Institutional Ethics Committee. After a thorough explanation of the study process, potential benefits, risks, and possible complications of surgery, written informed consent was obtained from all participants. Participants were informed that their participation would not affect their standard of care. They were also advised of their right to withdraw from the study without consequence.

All patient data was anonymized and stored securely to ensure confidentiality. Only authorized research personnel had access to the data. The study did not involve experimental interventions; all surgical procedures and follow-up care were conducted per standard clinical protocols. However, the additional measurements and examinations required for the study were clearly explained to participants.

## Results

The study included a total of 200 patients. The sample size was determined based on a power analysis conducted before the study, as described in the methodology section. The calculation assumed an effect size of 0.25, a 95% confidence interval, and 90% power, resulting in an estimated required sample size of 171 patients. The final target sample size was 200 patients, taking into account potential dropouts and increasing statistical power. The study included 108 right eyes and 92 left eyes. There was a relatively even distribution between the right and left eyes, with a slight predominance of the right eye (54%) compared to the left eye (46%). This distribution helped minimize potential bias related to eye laterality. The study population consisted of adults aged 40 and above, as per the inclusion criteria. The mean age of the study participants was 61.98 years, with a standard deviation of 7.686 years (range 42-84 years). This indicated that the study primarily focused on an older adult population, consistent with the typical age range for cataract development and surgery. The coefficient of variation for age was 12.40%, suggesting a moderate level of variability in the age distribution of the participants. The gender distribution was relatively balanced, with a slightly higher proportion of female participants (54%) compared to male participants (46%).

The mean axial length of the eyes included in the study was 23.0368 mm, with a standard deviation of 0.74316 mm. The coefficient of variation for axial length was 3.23%, indicating relatively low variability in this parameter among the study participants.

**Table 1** summarizes the key baseline characteristics of the study population. The coefficient of variation (CV) indicates the relative variability of each parameter within the sample.

**Table 1:** Baseline characteristics of the study patients (N=200)

Characteristic	Mean ± SD	Coefficient of Variation (%)
Age (years)	61.98 ± 7.686	12.40
Axial Length (mm)	23.0368 ± 0.74316	3.23
Baseline UCVA (logMAR)	0.6284 ± 0.23087	36.74
Baseline K1 (D)	42.7425 ± 1.46235	3.42
Baseline K2 (D)	43.3321 ± 1.43558	3.31
Baseline Astigmatism (D)	0.5586 ± 0.17503	31.33
Baseline HOA (μm)	0.1572 ± 0.0769	48.92
Baseline Pupil Size (mm)	4.2346 ± 1.33944	31.63

UCVA = Uncorrected Visual Acuity; K1 = Flat Keratometry; K2 = Steep Keratometry; HOA = Higher-Order Aberrations; SD = Standard Deviation

HOA measurements showed statistically significant changes from the first to the second time points (p < 0.001), although these were smaller than those of LOA. There was a slight increase from the first measurement (0.19 ± 0.06 μm) to the second (0.21 ± 0.08 μm), followed by a gradual decrease in the third (0.20 ± 0.07 μm) and fourth (0.19 ± 0.05 μm) measurements; however, the changes were statistically insignificant. The final HOA measurement returned to the initial value, suggesting that while there were temporary changes in HOAs, they stabilized by the fourth measurement. The smaller magnitude of changes in HOAs compared to LOAs indicated that the surgical intervention and healing process had a more pronounced effect on lower-order aberrations than on higher-order aberrations.

Lower-Order Aberrations (LOA): The changes in LOA were statistically significant (p < 0.001). This indicates that the substantial increase followed by a gradual decrease in LOA represented a genuine change in the eye’s optical quality over time.

Higher-Order Aberrations (HOA): Despite the smaller magnitude of changes, the variations in HOA over time showed statistically insignificant (p > 0.001). This suggested that even these subtle changes in higher-order aberrations represent actual fluctuations in the eye’s optical quality.

The parallel changes observed in astigmatism and lower-order aberrations suggest that the induced astigmatism was a major contributor to the overall change in optical quality. The temporary nature of these changes, with partial recovery by the fourth measurement, was encouraging for long-term visual outcomes. The relatively small changes in HOAs, with a return to baseline by the fourth measurement, suggested that the intervention did not have a significant long-term impact on higher-order optical quality. This was a positive finding, as increased HOAs could harm visual quality.

For most parameters, the most significant changes occurred between the first and second measurements, with a trend towards stabilization by the third and fourth measurements. This information could guide clinicians in determining the optimal timing for post-operative assessments and potential interventions. A comparison of the measurement methods is discussed in **Table 2**.

**Table 2:** Comparison of measurement methods

Parameter	S Method (Mean ± SD)	I Method (Mean ± SD)	P-value
K1 (D)	42.742± 1.46	42.73 ± 1.46	0.99
K2 (D)	43.33 ± 1.44	43.33 ± 1.44	0.99
Astigmatism (D)	0.56 ± 0.18	0.56 ± 0.18	0.99
LOA (μm)	0.29 ± 0.11	0.34 ± 0.16	0.005
HOA (μm)	0.19 ± 0.06	0.12 ± 0.08	0.005
Pupil Size (mm)	3.00 ± 0.00	5.47 ± 0.73	<0.001

K1 = Flat Keratometry; K2 = Steep Keratometry; LOA = Lower-Order Aberrations; HOA = Higher-Order Aberrations; SD = Standard Deviation

Significant differences were observed in aberration measurements and pupil size. These differences underscore the importance of considering the measurement method when interpreting results, particularly for parameters such as HOAs and pupil size, which can significantly impact visual quality assessments. A comprehensive comparison of measurements between S and I methods across all time points is mentioned in **Table 3**. Preoperative values for LOA and HOA are provided in **Table 4**.

**Table 3:** Comprehensive comparison of measurements between S and I methods across all time points (N=200)

Parameter	Time Point	S Method (Mean ± SD)	I Method (Mean ± SD)	P-value
K1 (D)	1st	42.74 ± 1.46	42.73 ± 1.46	0.295
	2nd	42.51 ± 1.46	42.52 ± 1.59	0.500
	3rd	42.78 ± 1.44	42.79 ± 1.43	0.533
	4th	42.79 ± 1.41	42.80 ± 1.40	0.90
K2 (D)	1st	43.33 ± 1.43	43.33 ± 1.44	.605
	2nd	43.45 ± 1.50	43.45 ± 1.50	.887
	3rd	43.52 ± 1.45	43.53 ± 1.46	.392
	4th	43.49 ± 1.44	43.49 ± 1.45	.837
Astigmatism (D)	1st	0.56 ± 0.18	0.56 ± 0.18	0.99
	2nd	0.71 ± 0.43	0.70 ± 0.34	0.99
	3rd	0.65 ± 0.43	0.66 ± 0.20	0.99
	4th	0.57 ± 0.22	0.57 ± 0.20	0.99
LOA (μm)	1st	0.28 ± 0.11	0.28 ± 0.11	0.140
	2nd	0.38 ± 0.16	0.37 ± 0.15	0.213
	3rd	0.32 ± 0.13	0.32 ± 0.13	.108
	4th	0.30 ± 0.12	0.30 ± 0.12	.106
HOA (μm)	1st	0.19 ± 0.06	0.12 ± 0.08	0.005
	2nd	0.21 ± 0.08	0.12 ± 0.06	0.001
	3rd	0.20 ± 0.07	0.12 ± 0.06	<0.001
	4th	0.19 ± 0.05	0.12 ± 0.07	<0.001

K1 = Flat Keratometry; K2 = Steep Keratometry; LOA = Lower-Order Aberrations; HOA = Higher-Order Aberrations; SD = Standard Deviation

**Table 4:** Pre-operation values for LOA and HOA

Variables	Mean	P value	Variables	Mean	P value
LOA (Preop)	0.29 ± 0.11		HOA (Preop)	0.157 ± 0.077 [0.19]	
2^nd^ measurement	0.38 ± 0.24	<0.001	2^nd^ measurement	0.167 ± 0.085 [0.2]	<0.001
3^rd^ measurement	0.32 ± 0.13	<0.001	3^rd^ measurement	0.162 ± 0.078 [0.19]	0.99
4^th^ measurement	0.30 ± 0.17	0.008	4^th^ measurement	0.155 ± 0.069 [0.18]	0.99

The measurement of lower-order aberrations showed notable differences between the S and I methods. At the first time point, the I method measured slightly higher LOA values compared to the S method (0.28 ± 0.16 μm vs. 0.29 ± 0.11 μm, p = 0.005). For subsequent time points, the S method consistently measured higher LOA values than the I method, with statistically significant differences (all p-values ≤ 0.001). The discrepancy between methods was most pronounced at the third and fourth time points, where the difference in mean LOA values was approximately 0.11 to 0.12 μm.

These significant differences in LOA measurements between S and I suggested that these techniques might not be interchangeable for assessing lower-order aberrations. The choice of method could have implications for clinical decision-making, particularly in cases where precise LOA measurements are crucial for treatment planning or outcome assessment.

The measurement of higher-order aberrations revealed consistent and statistically significant differences between the S and I methods: Across all four time points, the S method consistently measured higher HOA values than the I method. The differences were statistically significant at all time points (all p-values ≤ 0.005). The magnitude of the difference remained relatively stable across time points, with the S method measuring HOA values approximately 0.07 to 0.09 μm higher than the I method. The choice of measurement method could significantly impact the assessment of visual quality, as HOAs are known to affect visual performance and method-dependent sensitivity to these parameters. **Table 5** summarizes the key findings regarding the comparison between S and I methods. **Table 6** mentions the changes in Uncorrected Visual Acuity (UCVA) over time.

**Table 5:** Summary of key findings - comparison between S and I methods

Parameter	Consistency Between Methods	Significant Differences	Temporal Pattern Similarity
K1 (Flat Keratometry)	High	No	Similar
K2 (Steep Keratometry)	High	No	Similar
Astigmatism	High	No	Similar
Lower-Order Aberrations (LOA)	High	No (S > I)	Similar
Higher-Order Aberrations (HOA)	Low	Yes (S > I)	Different
Pupil Size	Very Low	Yes (I > S)	Different (S constant, I variable)

Note: “>” indicates consistently higher measurements

**Table 6:** Changes in Uncorrected Visual Acuity (UCVA) over time

Time Point	Mean UCVA (logMAR) ± SD	Median UCVA (logMAR)	p-value
Baseline	0.8584 ± 0.23087	0.80	-
At postop. day - 7	0.1907 ± 0.14265	0.18	<0.001
At postop. day 30	0.1550 ± 0.13792	0.15	<0.001
At postop. day 60	0.1526 ± 0.13578	0.15	<0.001

## Discussion

Cataract surgery has undergone significant evolution over the past few decades, transforming from a sight-restoring procedure to a refractive surgery aimed at optimizing visual outcomes. This transformation has been driven by advancements in surgical techniques, intraocular lens (IOL) technology, and diagnostic tools. As patient expectations continue to rise, there is an increasing focus on achieving excellent uncorrected visual acuity and quality of vision following cataract surgery.

The role of higher-order aberrations (HOAs) in visual quality has gained increasing attention. Unlike lower-order aberrations such as myopia, hyperopia, and astigmatism, which can be corrected with standard spherocylindrical lenses, HOAs represent more complex optical imperfections that can affect visual performance even in eyes with excellent visual acuity as measured by standard charts.

Advanced diagnostic technologies have revolutionized our ability to assess and analyze corneal properties and optical aberrations. Scheimpflug imaging, based on the principle of slit lamp biomicroscopy, provides detailed information about corneal topography, including both anterior and posterior surfaces. Complementing Scheimpflug imaging, ray-tracing technology has become a powerful tool for assessing ocular aberrations. By analyzing the path of light rays through the eye’s optical system, ray tracing provides a comprehensive assessment of both corneal and total ocular aberrations, offering insights into the complex interplay between different ocular structures in determining overall visual quality.

This study aimed to evaluate changes in higher-order aberrations (HOAs) following cataract surgery, as assessed by Scheimpflug imaging and ray-tracing technologies. The key findings suggest that corneal HOAs showed minimal changes, with slight increases immediately after surgery that tended to return to preoperative levels over time. Total ocular HOAs decreased significantly following surgery, particularly spherical aberration. The relative contribution of corneal HOAs to total ocular HOAs increased after surgery due to the significant reduction in internal aberrations.

The minor changes observed in HOAs suggest that modern small-incision cataract surgery has a relatively minimal impact on overall corneal shape. These changes diminish over time, indicating a gradual return to the preoperative corneal shape. This finding suggests that the biomechanical effect of small-incision surgery on the cornea is limited, primarily affecting the anterior surface near the incision site.

The significant reduction in total ocular HOAs appears to be a key factor in improving visual quality after cataract surgery. This reduction is primarily attributed to replacing the cataractous lens with an IOL rather than changes in corneal optics.

The increased relative contribution of corneal HOAs to total ocular HOAs postoperatively underscores the importance of considering corneal aberrations in surgical planning and IOL selection. As the cornea becomes the dominant source of aberrations post-surgery, strategies to address corneal HOAs may become increasingly relevant in optimizing visual outcomes.

The magnitude of the HOAs was also reduced, and the improvement in visual acuity outcomes suggests that aberrations play a role in patients’ visual quality. Other factors, such as neural adaptation and residual refractive error, may also significantly influence postoperative vision.

The significant reduction in total ocular HOAs, particularly spherical aberration, observed in our study is consistent with the findings of Marcos S et al. [4], who reported improvements in optical quality following cataract surgery with aspheric IOL implantation.

Our observation of minimal changes in corneal HOAs also aligns with their findings.

Other studies, such as Guirao A et al. [5], have noted the increased relative contribution of corneal HOAs to total ocular HOAs postoperatively, highlighting the importance of considering corneal aberrations in IOL design and selection.

Our study’s strengths include a comprehensive assessment of corneal and total ocular properties using advanced imaging technologies. Its longitudinal design with multiple postoperative time points allowed for analyzing changes over time. The large sample size included in this study provided robust statistical power. The comparison of two measurement technologies enhanced the reliability of the findings.

This study provides comprehensive insights into the changes in corneal curvature and higher-order aberrations following modern cataract surgery. The findings suggest that while small changes occur in corneal properties, these have minimal impact on visual outcomes. The significant improvement in vision appears to be primarily driven by replacing the cataractous lens with an IOL, particularly in reducing total ocular aberrations.

The excellent agreement between Scheimpflug imaging and ray-tracing aberrometry validates the use of these advanced technologies in assessing outcomes of cataract surgery.

Their ability to provide detailed analyses of corneal and total ocular properties offers valuable tools for clinical practice and research.

However, our study also had some limitations, including a lack of assessment of contrast sensitivity and other visual quality measures beyond visual acuity, as well as a limited analysis of the impact of different IOL designs on outcomes. This was a single-center study, which may have limited its generalizability. The relatively short follow-up period (2 months) might preclude analysis of long-term changes.

Future studies should extend the follow-up period to assess the long-term stability of corneal properties and visual outcomes following cataract surgery. This would provide valuable insights into the durability of surgical outcomes and the potential for late changes in corneal shape or optical properties.

Research into the impact of newer IOL designs, such as extended depth of focus or accommodating IOLs, on corneal properties and HOAs would be valuable. Additionally, studies exploring the potential for customized IOLs based on individual corneal aberration profiles could improve visual outcomes.

Diagnosing personalized surgical approaches, such as customized incision placement or corneal relaxing incisions based on preoperative corneal analysis, could potentially enhance outcomes for patients with significant preoperative corneal irregularities or astigmatism.

## Conclusion

In conclusion, as evaluated in this study, modern cataract surgery is a safe and effective procedure that significantly improves visual acuity while maintaining corneal integrity. The detailed understanding of corneal and optical changes provided by this research contributes to the ongoing refinement of surgical techniques and technologies in cataract surgery.

## Presentation in an event

The paper has not been presented at any academic conference.
